# Chronic disease relapses: A cross-sectional study of the associated factors and socioeconomic inequalities during the COVID-19 pandemic in Peru

**DOI:** 10.1371/journal.pone.0274697

**Published:** 2022-09-16

**Authors:** Fabriccio J. Visconti-Lopez, Akram Hernández-Vásquez, Dustin M. Solorzano-Salazar, Diego Azañedo

**Affiliations:** 1 Universidad Peruana de Ciencias Aplicadas, Lima, Peru; 2 Centro de Excelencia en Investigaciones Económicas y Sociales en Salud, Vicerrectorado de Investigación, Universidad San Ignacio de Loyola, Lima, Peru; 3 Universidad Científica del Sur, Lima, Peru; University of Copenhagen: Kobenhavns Universitet, DENMARK

## Abstract

**Objectives:**

To investigate the prevalence, associated factors and socioeconomic inequalities in chronic disease relapses (CDR) during 2020 in Peru.

**Methods:**

A secondary analysis was made of the National Household Survey on Living Conditions and Poverty (ENAHO) 2020. Participants older than 18 years who suffered from a chronic disease and with information about the occurrence of a CDR in the last 4 weeks prior to the survey were included. Adjusted prevalence ratios (aPRs) were estimated to determine the associated factors. Socioeconomic inequality in CDR was estimated using concentration curves (CC) and the Erreygers concentration index (ECI).

**Results:**

Data from 38,662 participants were analyzed; the prevalence of CDR in the last 4 weeks prior to the survey was 16.5% (95% CI: 15.8–17.2). Being female (aPR 1.29; 95% CI: 1.21–1.37), with regards to being male; being 30–39 (aPR 1.22; 95% CI: 1.05–1.42), 40–49 (aPR 1.29; 95% CI: 1.12–1.48), 50–59 (aPR 1.60; 95% CI: 1.41–1.82), and 60 years or older (aPR 1.80; 95% CI: 1.58–2.04), compared to 18–29; reaching up to primary (aPR 1.18; 95% CI: 1.07–1.31), or secondary education (aPR 1.13; 95% CI: 1.02–1.24), in contrast to tertiary education; presenting some physical, psychological or cognitive limitation (aPR 1.33; 95% CI: 1.21–1.46), with respect to experiencing no limitations; and being affiliated to a health insurance (aPR 1.18; CI 95%: 1.09–1.29), opposed to not having health insurance; were associated with a higher probability of CDR. Residing in the natural region of the coastal area (aPR 0.83; 95% CI: 0.74–0.92) was associated with a lower probability of relapse compared to residing in the jungle area. In people with limitations and residents of the jungle areas, the prevalence of CDR was concentrated in those with higher per capita spending.

**Conclusions:**

Approximately 1 in 6 Peruvians with chronic diseases had a relapse within the last 4 weeks prior to the survey of 2020 and certain geographic and sociodemographic factors were found to be associated with CDR. It was also found that a higher concentration of CDR was observed in the population with the highest per capita spending with some limitations, as well as in residents of the jungle, implying the need for appropriate policy interventions that address CDR with a special focus on these populations.

## Introduction

Chronic diseases are a group of long-term pathologies resulting from genetic, physiological, environmental, and behavioral factors [1]. These diseases are estimated to account for approximately 41 million deaths worldwide, which is equivalent to 70% of all annual deaths globally [1]. People with chronic diseases should receive adequate control, treatment and rehabilitation to contribute to their stabilization and prevent future complications [2]. However, in some cases, patients may experience exacerbations or relapses, which can place them at risk of functional deterioration and compromise their prognosis [3]. Therefore, the management of these pathologies requires long-term treatment and control, with good adherence and continuous evaluation.

Chronic diseases, such as cardiovascular diseases, chronic obstructive pulmonary disease, cancers, Alzheimer’s and other dementias, and diabetes mellitus, were among the top 10 causes of death worldwide in 2019 [4]. The incidence of these diseases increases with age and is more prevalent in low- and middle-income countries, such as in Latin America and the Caribbean (LAC) [5]. The impact on low- and middle-income countries is so great that the risk of dying from a chronic disease is twice that of high-income countries [6]. Likewise, approximately three-quarters of all deaths from chronic diseases and 82% of the 16 million people who died prematurely or before the age of 70 years occur in low- and middle-income countries [7]. In Peru, 69% of all annual deaths are caused by chronic diseases; moreover, the probability of premature death from these disorders is approximately 15% [8].

In the context of COVID-19, access to health services has been very limited, with a reduction in the use of health services at different levels of care [9] and having a great effect on chronic diseases [10, 11]. This access to health services has had a greater impact in developing countries, such as LAC countries, which have a higher proportion of the population living in poverty, a higher population density in rural and marginal areas, and an increasing prevalence of chronic disease compared to high-income countries [12, 13]. It has been reported that people with chronic diseases have difficulties attending medical visits, which likely affects new diagnoses and treatments [9].

At the beginning of June 2021, Peru was one of the countries most affected by the COVID-19 pandemic with 5,551 COVID-19 deaths per million inhabitants, being the highest figure globally; furthermore, it is the country with the highest fatality rate in the world (9.4%) [14, 15]. This great health impact on the country has caused a collapse in the health system, which could lead to a greater number of chronic disease relapses (CDR) due to the increased demand and saturation of health services for the control of these diseases [16]. The impact of the COVID-19 pandemic on the population with chronic diseases has been poorly studied in low- and middle-income countries. In fact, to date, no population-based studies assessing the prevalence of CDR with related socioeconomic factors or inequalities in low- and middle-income countries have been identified. For this reason, the objective of this study was to investigate the prevalence, associated factors and socioeconomic inequalities in CDR during 2020 in Peru.

## Materials and methods

### Design and study population

This cross-sectional study was carried out using publicly available data from the National Household Survey on Living Conditions and Poverty (ENAHO) of the year 2020. The methodological details of the survey have been described elsewhere [17]. Briefly, the ENAHO is a survey developed and executed by the National Institute of Statistics and Informatics (INEI) of Peru with national representation and collects socioeconomic information from the members of Peruvian households [17]. It is a survey with a probabilistic, area, stratified, multistage, and independent sampling carried out through face-to-face interviews. The sample size was 37,103 homes, 23,895 homes corresponding to the urban area, and 13,208 homes to the rural area [17]. Data collection of the ENAHO 2020 during the period of social isolation was carried out by telephone interviews (from March 16 to September 30, 2020) and later by face-to-face interviews (from October to December 2020) supplemented with telephone interviews [17]. The technical documents related to the design, instruments, procedures, and manuals of ENAHO 2020, are published freely in the "Documentación" tab at the following link http://iinei.inei.gob.pe/microdatos/.

**Fig 1:**
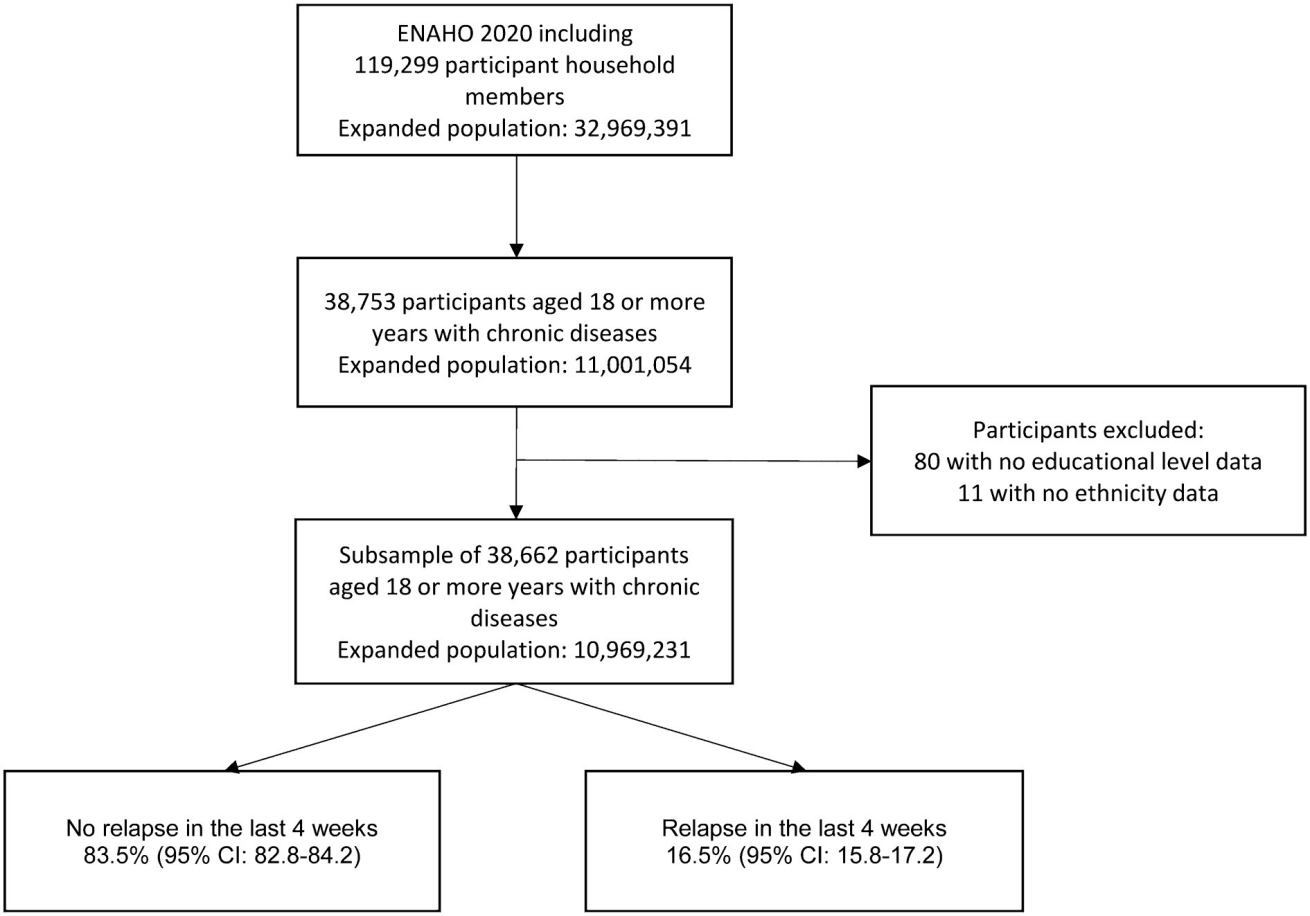
Flowchart of participants included in the study.

The present study included a subsample of 38,662 Peruvian adult participants aged 18 or over who answered the following question affirmatively: Do you suffer from any chronic illness or discomfort? Fig 1 shows the flowchart of the study participants.

### Variables and measurements

The dependent variable was having presented any CDR in the last four weeks prior to the survey reported by the answer to the question: “In the last 4 weeks, did you present any relapse of chronic disease…?”. The responses were classified as 1 "Yes, presented" and 0 "Did not present."

The following independent variables were included: sex (categorical: male, female), age group (categorical: 18–29, 30–39, 40–49, 50–59, 60 or more years), educational level (categorical: up to primary education, secondary education, or tertiary education), marital status (categorical: without partner, with partner), ethnic self-identification (categorical: non-native, native), presence of some physical or psychological or cognitive limitation (categorical: no, yes), quintiles of per capita household expenditure (categorical: poorest, poorest, middle, rich, richest), affiliation to health insurance (categorical: no, yes), natural region of residence (categorical: coastal areas, highland areas, jungle areas) and area of residence (categorical: urban areas, rural areas). In the ethnicity variable, the native category is made up of people who identify themselves as belonging to an indigenous ethnic group in Peru, while the non-native category is made up of people who identify themselves as white, “mestizo”, black, “zambo”, mulatto or Afro-Peruvian people or other groups that are not recognized as natives. We used per capita household expenditure as an independent variable considering that this variable is officially used in Peru by the National Institute of Statistics and Informatics for the calculation of poverty [18]. These independent variables were selected due to their relationship with the burden of treatment for chronic diseases, following the conceptual framework proposed by Sav *et al*. [19] and factors associated with the non-use of health services in the Peruvian population [20].

### Statistical analysis

In the descriptive analysis, the sociodemographic characteristics of the study population were described using weighted frequencies and proportions. In addition, CDR was described according to the sociodemographic characteristics of the study population. Inferential analysis of the factors associated with CDR in Peruvian adults was performed using a generalized linear model of the Poisson family and logarithmic link to obtain crude and adjusted prevalence ratios (PR) together with their 95% confidence intervals (CI) [21, 22]. Variables that obtained a p-value <0.20 in the crude analysis were included in the adjusted analysis [23]. The presence of multicollinearity between the independent variables was previously determined using the variance inflation factor (VIF).

To measure socioeconomic inequalities in CDR by sex, the presence of limitations, natural region of residence, and area of residence, concentration curves (CC) and the Erreygers concentration index (ECI) were estimated. As for the CC, these describe the relationship between the cumulative percentage of CDR in the population according to their level of wealth as measured by per capita household expenditure (X-axis), and the cumulative percentage of CDR (Y-axis) with the diagonal line of equality. Inequality is estimated according to the location of the CC and the further it is from the diagonal line of equality, the greater the inequality [24]. If the CC is located below the diagonal line of equality, there is a greater burden of CDR in people from households with higher per capita spending and if the CC is located above the diagonal line of equality there is a greater burden of relapses in people from households with the highest per capita spending. The ECIs were estimated using the command "conindex" because the dependent variable is dichotomous in nature [24]. Briefly, the ECI values range between -1 and 1 [24], with positive values indicating a greater burden of CDR in people from households with higher per capita spending and negative values indicating that the burden of relapses would be in people from households with lower per capita spending.

All analyses were performed using the statistical program Stata^®^ version 14.2 (Stata Corporation, College Station, Texas, USA) and the level of significance was considered as p <0.05. Likewise, the sample weights and the complex design of the ENAHO 2020 were used for all estimates. In addition, the analyses for the subpopulation of interest were specified using the "subpop" option of the Stata program.

### Ethical considerations

The approval of an ethics committee was not requested as it is an analysis of secondary data that is in the public domain and does not allow the respondents to be identified. All ENAHO 2020 data are completely anonymized and respondents provided informed consent prior to their participation. The databases used are freely accessible on the INEI web portal (http://iinei.inei.gob.pe/microdatos/). The information can be obtained by entering the “Consulta por Encuestas” tab and selecting the “ENAHO Metodología ACTUALIZADA” data.

## Results

Of a total of 38,662 participants who suffer from a chronic disease, 16.5% (95% CI: 15.8–17.2) presented a relapse of their disease in the last 4 weeks in 2020. Most of the participants belonged to the female sex (57.3%), to the age group of 60 years and over (34.3%), had reached an educational level up to primary education (35.4%), and had a partner (58%). Likewise, the majority self-rated as non-native (72.1%); 90% of the participants did not present any physical, psychological or cognitive limitation and 21.5% were classified as rich, according to the quintiles of per capita household expenditure. Similarly, 78.4% of those surveyed had health insurance. The highest proportion of participants came from the natural region of the coastal areas (59.6%), and 82.7% of those surveyed resided in urban areas (Table 1).

**Table 1:** Characteristics of the participants included in the study.

Characteristics	n (%)	Chronic disease relapse		P value
		No	Yes	
		% (95% CI)	% (95% CI)	
Overall	38,662 (100)	83.5 (82.8–84.2)	16.5 (15.8–17.2)	
Sex				
Male	16,524 (42.7)	85.9 (85.0–86.7)	14.1 (13.3–15.0)	<0.001
Female	22,138 (57.3)	81.8 (80.9–82.7)	18.2 (17.3–19.1)	
Age groups				
18–29 years	5,061 (14.7)	90.2 (89.0–91.2)	9.8 (8.8–11.0)	<0.001
30–39 years	4,835 (12.8)	87.0 (85.6–88.4)	13.0 (11.6–14.4)	
40–49 years	7,032 (18.1)	86.0 (84.9–87.2)	14.0 (12.8–15.1)	
50–59 years	8,063 (20.1)	82.3 (81.0–83.6)	17.7 (16.4–19.0)	
60 or more years	13,671 (34.3)	78.8 (77.6–79.9)	21.2 (20.1–22.4)	
Education level				
Tertiary education	11,153 (29.3)	87.5 (86.4–88.4)	12.5 (11.6–13.6)	<0.001
Secondary education	12,319 (35.3)	84.9 (83.8–85.9)	15.1 (14.1–16.2)	
Up to primary education	15,190 (35.4)	78.9 (77.8–80.0)	21.1 (20.0–22.2)	
Partner				
Without partner	15,466 (42.0)	84.1 (83.2–85.0)	15.9 (15.0–16.8)	0.064
With partner	23,196 (58.0)	83.1 (82.3–83.9)	16.9 (16.1–17.7)	
Ethnicity				
Non-native	27,241 (72.1)	84.1 (83.3–84.9)	15.9 (15.1–16.7)	0.007
Native	11,421 (27.9)	82.1 (80.7–83.4)	17.9 (16.6–19.3)	
Has any physical, psychological or cognitive limitation				
No	34,452 (90.0)	84.5 (83.7–85.2)	15.5 (14.8–16.3)	<0.001
Yes	4,210 (10.0)	75.3 (73.1–77.3)	24.7 (22.7–26.9)	
Quintiles of per capita household expenditure				
Poorest	7,734 (16.6)	83.5 (82.1–84.7)	16.5 (15.3–17.9)	0.941
Poor	7,695 (19.3)	83.3 (81.9–84.5)	16.7 (15.5–18.1)	
Middle	7,535 (21.2)	83.5 (82.1–84.8)	16.5 (15.2–17.9)	
Richer	7,822 (21.5)	84.0 (82.6–85.4)	16.0 (14.6–17.4)	
Richest	7,876 (21.4)	83.4 (82.0–84.8)	16.6 (15.2–18.0)	
Has health insurance				
No	7,521 (21.6)	87.2 (86.1–88.3)	12.8 (11.7–13.9)	<0.001
Yes	31,141 (78.4)	82.5 (81.7–83.3)	17.5 (16.7–18.3)	
Natural region of residence				
Jungle areas	7,136 (10.6)	81.4 (79.9–82.9)	18.6 (17.1–20.1)	<0.001
Highland areas	13,524 (29.8)	80.1 (78.7–81.5)	19.9 (18.5–21.3)	
Coastal areas	18,002 (59.6)	85.6 (84.6–86.5)	14.4 (13.5–15.4)	
Residence area				
Rural	12,109 (17.3)	78.7 (77.4–80.0)	21.3 (20.0–22.6)	<0.001
Urban	26,553 (82.7)	84.5 (83.7–85.3)	15.5 (14.7–16.3)	

Estimates include the weights and ENAHO 2020 sample specifications.

The P value was calculated using the Rao-Scott Chi-squared.

CI: confidence interval.

Significant differences were found between the presence of relapses due to chronic diseases according to the categories of variables: sex (p <0.001), age group (p <0.001), educational level (p <0.001), ethnicity (p = 0.007), presence of some physical, psychological or cognitive limitation (p <0.001), health insurance (p <0.001), natural region of residence (p <0.001) and area of residence (p <0.001). Thus, it was found that approximately 20% of women and at least 20% of people aged 60 or over had a CDR. On the other hand, at least 21.1% of people with an educational level up to primary education and 17.9% of the people who self-described as natives had some episode of CDR. Similarly, 24.7% of those who presented some physical, psychological or cognitive limitation and 17.5% of respondents who had health insurance had had at least one episode of CDR. Likewise, approximately 19.9% of people who reside in the natural region of the highland areas and 21.3% of people who reside in rural areas had had CDR in the last 4 weeks (Table 1).

In the crude model, the factors associated with having a higher probability of CDR were being female compared to male; being 30–39, 40–49, 50–59, or 60 years or older compared to 18–29 years; having an educational level up to primary, and secondary education, compared to those with a tertiary education level; self-identifying as native compared to non-natives; presenting some physical, psychological or cognitive limitation compared to those who do not and having health insurance compared to those who do not. On the other hand, the factors associated with having a lower probability of CDR were: residing in the natural region of the coastal areas compared to those residing in the jungle areas and residing in urban areas in comparison with those residing in rural areas (Table 2).

**Table 2:** Crude and adjusted prevalence ratios of chronic disease relapses.

Variable	Crude		Adjusted	
	PR (95% CI)	P value	PR (95% CI)	P value
Sex				
Male	Ref.		Ref.	
Female	1.29 (1.21–1.37)	<0.001	1.29 (1.21–1.37)	<0.001
Age groups				
18–29 years	Ref.		Ref.	
30–39 years	1.32 (1.14–1.54)	<0.001	1.22 (1.05–1.42)	0.010
40–49 years	1.42 (1.24–1.63)	<0.001	1.29 (1.12–1.48)	<0.001
50–59 years	1.80 (1.60–2.04)	<0.001	1.60 (1.41–1.82)	<0.001
60 or more years	2.17 (1.92–2.44)	<0.001	1.80 (1.58–2.04)	<0.001
Education level				
Tertiary education	Ref.		Ref.	
Secondary education	1.21 (1.09–1.33)	<0.001	1.13 (1.02–1.24)	0.020
Up to primary education	1.68 (1.54–1.84)	<0.001	1.18 (1.07–1.31)	0.001
Partner				
Without partner	Ref.		Ref.	
With partner	1.06 (1.00–1.13)	0.065	0.99 (0.93–1.06)	0.842
Ethnicity				
Non-native	Ref.		Ref.	
Native	1.13 (1.03–1.23)	0.006	0.92 (0.84–1.01)	0.076
Has any physical, psychological or cognitive limitation				
No	Ref.		Ref.	
Yes	1.59 (1.46–1.74)	<0.001	1.33 (1.21–1.46)	<0.001
Quintiles of per capita household expenditure				
Poorest	Ref.		Not incluided	
Poor	1.01 (0.91–1.12)	0.841	--	
Middle	1.00 (0.89–1.12)	0.988	--	
Richer	0.97 (0.86–1.09)	0.561	--	
Richest	1.00 (0.89–1.12)	0.995	--	
Has health insurance				
No	Ref.		Ref.	
Yes	1.37 (1.25–1.49)	<0.001	1.18 (1.09–1.29)	<0.001
Natural region of residence				
Jungle areas	Ref.		Ref.	
Highland areas	1.07 (0.96–1.19)	0.210	1.06 (0.95–1.18)	0.322
Coastal areas	0.78 (0.70–0.86)	<0.001	0.83 (0.74–0.92)	0.001
Residence area				
Rural	Ref.		Ref.	
Urban	0.73 (0.67–0.79)	<0.001	0.91 (0.83–1.01)	0.065

Estimates include the weights and ENAHO 2020 sample specifications.

PR: prevalence ratio; CI: confidence interval.

In the adjusted multivariate model, the factors associated with having a higher probability of CDR were being female (aPR 1.29; 95% CI: 1.21–1.37; p <0.001) compared to those of the male sex; being 30–39 years old (aPR 1.22; 95% CI: 1.05–1.42; p = 0.010), being 40–49 years old (aPR 1.29; 95% CI: 1.12–1.48; p <0.001), being 50–59 years old (aPR 1.60; 95% CI: 1.41–1.82; p <0.001) and being 60 years of age or older (aPR 1.80; 95% CI: 1.58–2.04; p <0.001) compared to those aged 18–29 years; having a secondary educational level (aPR 1.13; 95% CI: 1.02–1.24; p = 0.020) and having an educational level up to primary education (aPR 1.18; 95% CI: 1.07–1.31; p = 0.001) compared to tertiary education; presenting some physical, psychological or cognitive limitation (aPR 1.33; 95% CI: 1.21–1.46; p <0.001) compared to those who do not and having health insurance (aPR 1.18; 95% CI: 1.09–1.29; p <0.001) compared to those without insurance. On the other hand, the factor associated with having a lower probability of CDR in the adjusted model was residing in the natural region of the coastal areas (aPR 0.83; 95% CI: 0.74–0.92; p = 0.001) compared to those who reside in the jungle areas (Table 2).

**Fig 2:**
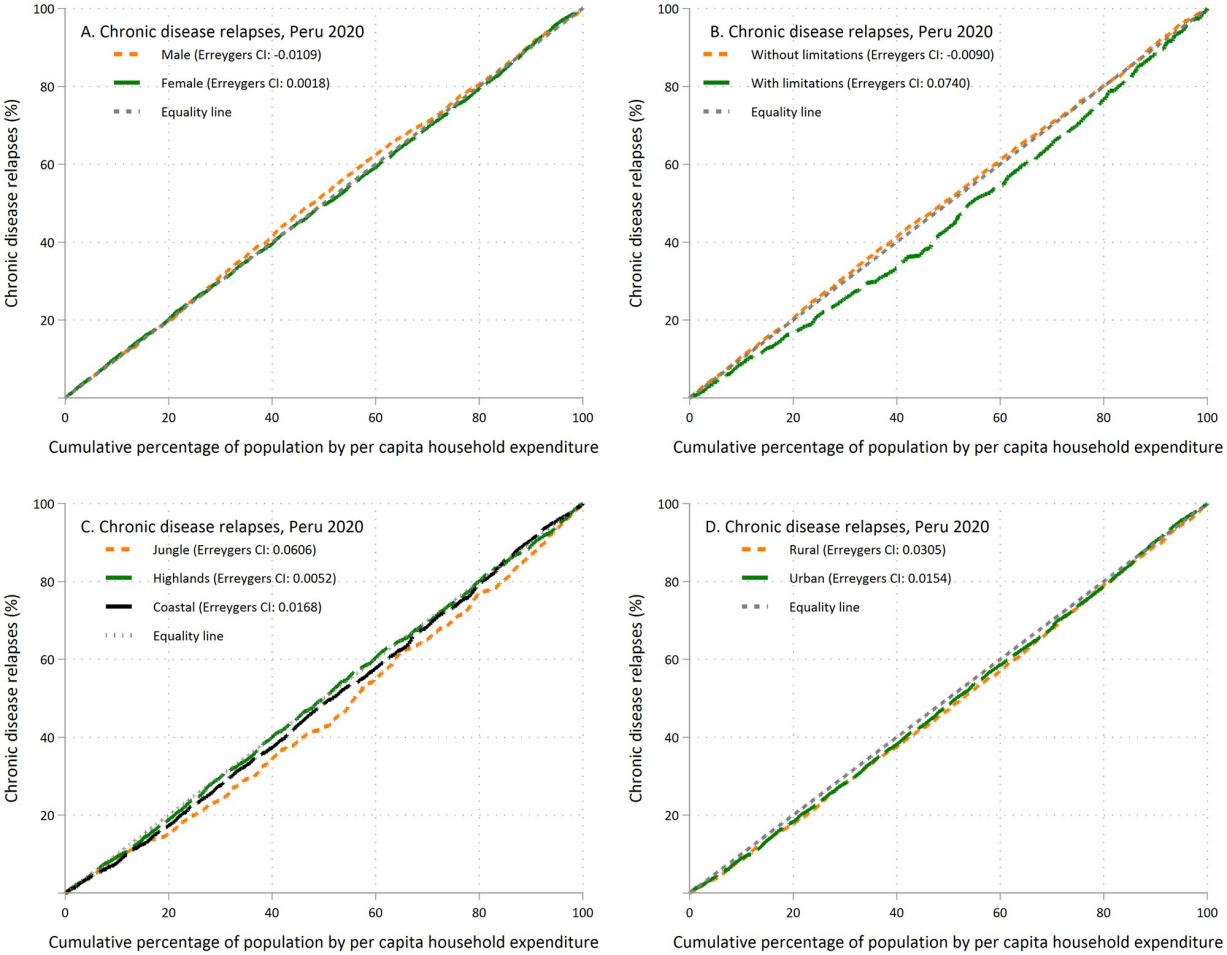
Concentration curves of chronic diseases relapses.

There were no inequalities in CDR according to the variables of sex and area of residence (Fig 2A and 2D). However, the CC of people with some limitations fell below the equality line, that is, the CDRs were concentrated in people with the highest per capita spending (Fig 2B). Likewise, concerning the region of residence, inequalities were identified in the people who reside in the jungle areas in which CDRs were concentrated among those with the highest per capita spending (Fig 2C).

## Discussion

The present study sought to evaluate the prevalence of CDR, associated factors, and related socioeconomic inequalities. It was found that approximately 1 in 6 people who suffer from a chronic disease had a CDR in the last 4 weeks. Likewise, being female, being 60 years of age or older, having an educational level up to elementary school, presenting some physical, psychological, or cognitive limitation, and having health insurance were associated with a higher probability of CDR. Residing in the natural region of the coast was associated with a lower probability of relapses compared to residing in the jungle. Finally, a higher concentration of CDR was found in the population with the highest per capita spending with some limitations and residents of the jungle, which would indicate the presence of inequalities in CDR according to socioeconomic conditions. It should be noted that the study of CDR is particularly important for public health in developing countries, such as those that make up LAC, because of the barriers to access health care brought about by the COVID-19 pandemic which may have seriously affected patients with chronic diseases.

This study found that being female was associated with a higher likelihood of CDR. Indeed, it has been shown that women are more likely to be diagnosed and receive care for non-communicable diseases due to a greater awareness of the signs and symptoms of these diseases and, consequently, an increase in seeking care in health centers [25]. However, barriers in access to health services have been accentuated with the pandemic, such as having less free time due to family responsibilities, a reduction in the ability to pay for health care due to shorter work hours, higher unemployment, decreased economic income, and the fear of being infected by COVID-19 [26]. In this way, the delay in the care of patients with chronic diseases may affect the probability of presenting CDR. Likewise, a survey on health care carried out during the last two months of 2020 in women residing in the United States reported that 46% of women with poor health status avoided preventive services, and 32% ignored undergoing diagnostic tests or performing recommended treatments [27]. Plus, it should be noted that in this survey teleconsultation was carried out for the management of chronic conditions, and this type of consultation doubled due to symptoms related to COVID-19 [27].

Being older was associated with a higher probability of CDR. Aging is a risk factor for the incidence and severity of chronic diseases as well as for CDR [28–30]. Moreover, it is reported that after the age of 35, people are more sedentary and obese, and this is associated with a higher prevalence and a greater number of chronic diseases [31]. It is of note that this setting has worsened due to the restrictions of the pandemic, such as social immobilization, contributing to an increase in sedentary lifestyle and obesity and, therefore, to a higher risk of CDR [32–35]. Nevertheless, during the pandemic, it was evidenced that relapses in patients with chronic obstructive pulmonary disease (COPD) decreased by more than 50% compared to previous years due to follow-up, care, and adherence to preventive measures; this is especially relevant since this group is at high risk for severe COVID -19 infection [36]. This suggests that COPD can be potentially manageable with appropriate measures in older patients, even in times of pandemic. This kind of approach should be investigated for the management of other chronic conditions in older adults to decrease the rate of relapses.

Having some physical, psychological, or cognitive limitation was found to be significantly related to presenting a relapse. In general, patients with limitations or disabilities report worse health condition and greater dissatisfaction with their lives, reflected by unhealthy attitudes such as greater intake of alcohol, tobacco, and less physical activity [31]. As mentioned above with older patients, this type of harmful attitude has worsened due to the pandemic, also increasing the risk of obesity and associated chronic diseases in patients with limitations. Lastly, patients with any type of limitation require greater use of health services; yet, these are often hampered by the limited capacity of the health institution due to COVID-19 for specific management of these patients, making treatment more expensive and resulting in poor adherence, and worsening the quality of life [37–39]. Due to the pandemic, this group of people had greater difficulty in accessing health services due to restrictions, scarce human resources, and the lack of operational and logistical capacity necessary for their care, all of which may have significantly reduced care in this population and increased the occurrence of CDRs [39].

This study reported that being insured was associated with a higher prevalence of CDR. In this regard, having health insurance is associated with better use of health services and favorable health outcomes [40–42]. Similarly, it has been documented that the presence of chronic diseases in the family environment is related to a greater search for care or insurance [43]. However, in Peru, there are barriers to access to health services, such as economic and infrastructural incapacity and inadequate mobilization that affect both the care and the availability of drugs. These problems have been exacerbated by the COVID-19 pandemic, particularly complicating patients with chronic diseases [44]. Thus, despite having health insurance, the delay or rejection of medical care due to fear of COVID-19 infection could have been directly or indirectly linked to an increase in excess deaths by COVID-19 [45, 46]. On the other hand, cultural characteristics typical of some sectors of the population could have also influenced the higher prevalence of CDR despite having health insurance. For example, a study carried out in the highlands of Peru showed that rural towns (n = 619) used more traditional treatments than urban towns (n = 156) (5.7% vs. 3.8%, respectively) and attended health establishments less often (86.8% vs. 98.5% attendance, respectively) [47]. This attitude can be detrimental by delaying the necessary treatment in patients with chronic diseases, increasing the probability of CDR. Therefore, despite having health insurance, there are other barriers at the level of the health system in Peru, such as access, infrastructure, and provision of medicines, as well as cultural barriers at a personal level. All of these can greatly affect the probability of having CDR and may have been accentuated by the COVID-19 pandemic. These barriers need to be addressed as a priority by the Peruvian Ministry of Health.

It was found that a lower educational level (high school or elementary) had a greater association with CDR. Other studies have reported associations between a low educational level and risk factors for the development of chronic diseases and their relapses [48], such as increased smoking, poor nutrition, low levels of physical activity and illiteracy in health [49]. By contrast, having a higher educational level is significantly associated with greater knowledge regarding chronic diseases [50]. In addition, a higher level of education is associated with higher socioeconomic status, allowing these individuals to have greater access to health services [51]. For this reason, CDRs may be found in a higher proportion of patients with a low educational level since they may be exposed to risky behaviors and decreased access to health services. It is noteworthy that in our study, no association was identified between the per capita spending quintiles and CDR, although this variable as well as educational level would be expected to be associated with this outcome. Future studies should address new hypotheses about the individual role and interactions of these variables on CDRs in the Peruvian context.

Living in the natural region of the coast compared to residing in the jungle was associated with a lower probability of CDR. This may be due to the greater availability of both public and private health services and also of health professionals on the coast. In the 2018 Health Situation Analysis, it was shown that from 2002 to 2016, outpatient care on the coast had increased by 50%, while in the jungle it had remained almost unchanged [52]. Furthermore, the existence of geographical barriers to health care influences differences in health conditions in the various natural regions. For example, the mortality rates of children under five years of age between 2013 and 2017 varied with the location of residence, being lower (26%) on the coast and higher in the highlands (39%) and jungle (42%). [53, 54]. Given this, it is important to facilitate and guarantee timely care to patients with CDR in the Peruvian jungle; likewise, new health interventions should be prioritized in this region.

Regarding the analysis of inequalities, CDR was concentrated among people with some limitations with the highest per capita spending. These inequalities may be due to difficulties in achieving access to drugs and medical checkups in patients with chronic diseases, which has been documented during the restrictions at the beginning of the pandemic [55]. This could have generated problems in adherence to treatment, and consequently, inadequate control and exacerbation of the chronic disease. Similarly, various studies have reported that the consumption of processed foods and alcoholic beverages increased during the mandatory quarantine [56, 57]. All of this could have caused a worsening of pre-existing chronic conditions in the Peruvian population and may have particularly affected individuals with limitations since they often cannot attend a medical consultation independently and depend on the support of a family member.

On the other hand, people with the highest per capita spending in the jungle concentrated the prevalence of CDR. This may be explained because as a region of difficult access, there may have had a shortage of drugs for chronic conditions in the jungle. Furthermore, the jungle is a region with few health professionals, and it also showed high figures in the contagion and mortality rates during the first months of the pandemic [58]. Likewise, it should be taken into account that the wealthier population was more able to comply with the mandatory quarantine, including a restriction on medical checkups because they had the opportunity to carry out remote work and have the economic solvency to cover basic needs. However, more studies related to the subject are required to elucidate these hypotheses.

This study has inherent limitations due to its design. One limitation is that it does not allow determining causality due to the lack of temporality because of its cross-sectional design. Additionally, there is the possibility of memory and information bias since the measurement of the variables of interest was self-reported based on the questions “suffer from a chronic disease” and “having a CDR in the last 4 weeks”. Likewise, since it was a secondary data analysis, only the variables collected in the survey could be included in the model, and there may be other influencing factors, such as the attitudes toward seeking medical care due to the disease, or the perception of chronic diseases and their relapses, which were not analyzed due to their unavailability in the database. However, the ENAHO is a nationally representative survey that allows the study of CDR and the identification of the associated factors. This study can serve as a basis for future prospective research on this topic.

In conclusion, approximately 1 in 6 Peruvians with chronic diseases had a relapse within the last 4 weeks prior to the survey of 2020 and certain geographic and sociodemographic factors were found to be associated with CDR. The presence of CDR could generate functional deterioration and compromise the prognosis of patients with chronic diseases. Therefore, it is necessary to formulate and implement public health measures that address this problem, such as, for example, improving the surveillance of chronic diseases and their complications, and carrying out campaigns to promote the health of the population taking into account the associated factors as well as the inequalities that exist within this area to reduce the gaps with the aim of decreasing CDR. This is of vital importance, especially in low- and middle-income countries in which the COVID-19 pandemic has had a greater impact on health systems.
